# Supplementary material for: Sphingosine-1-Phosphate Receptor Type 4 (S1P4) Is Differentially Regulated in Peritoneal B1 B Cells upon TLR4 Stimulation and Facilitates the Egress of Peritoneal B1a B Cells and Subsequent Accumulation of Splenic IRA B Cells under Inflammatory Conditions
Source: Int J Mol Sci. 2021 Mar 27;22(7):3465. doi: 10.3390/ijms22073465 (PMC8037865; doi:10.3390/ijms22073465)
Supplement: Supplementary file 1 [file ijms-22-03465-s001.pdf]

Supplementary Material to „Sphingosine-1-phosphate receptor type 4 ($S1P_4$) is differentially regulated on peritoneal B1 cells upon TLR4 stimulation and facilitates egress of peritoneal B1a cells and subsequent accumulation of splenic IRA B cells under abdominal inflammatory conditions“

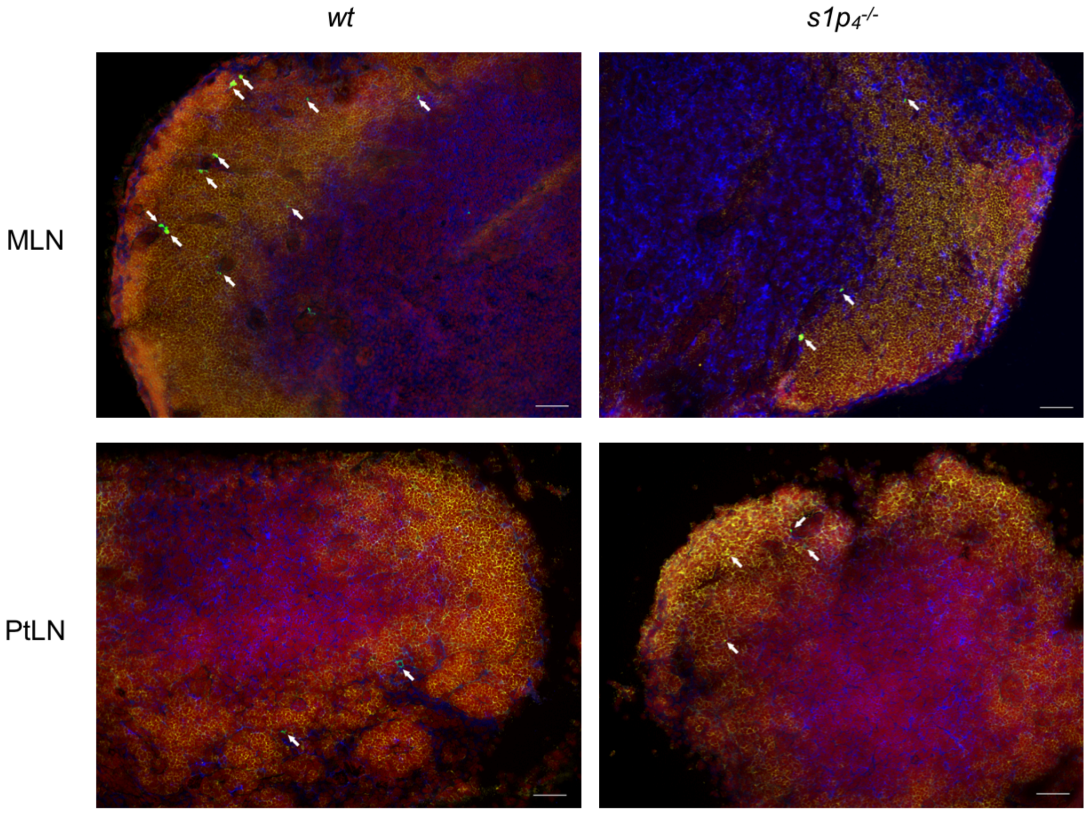

Supplementary Figure 1. Distribution of labeled peritoneal B cells in lymph nodes after adoptive peritoneal transfer and repeated LPS-stimulation.

72 hours after adoptive transfer of labelled peritoneal cells and repeated LPS pulses, the mesenterial (MLN), and parathymic (PtLN) lymph nodes were harvested. Sections (3 µm) were stained with anti-B220 (yellow), anti-CD4 (blue), anti-CFSE (green) and Draq5 (red). Labeled B cells were identified as $CFSE^+$ $B220^+$ (white arrows). Representative images for 5 mice per group are shown (white bar equals 50 µm).

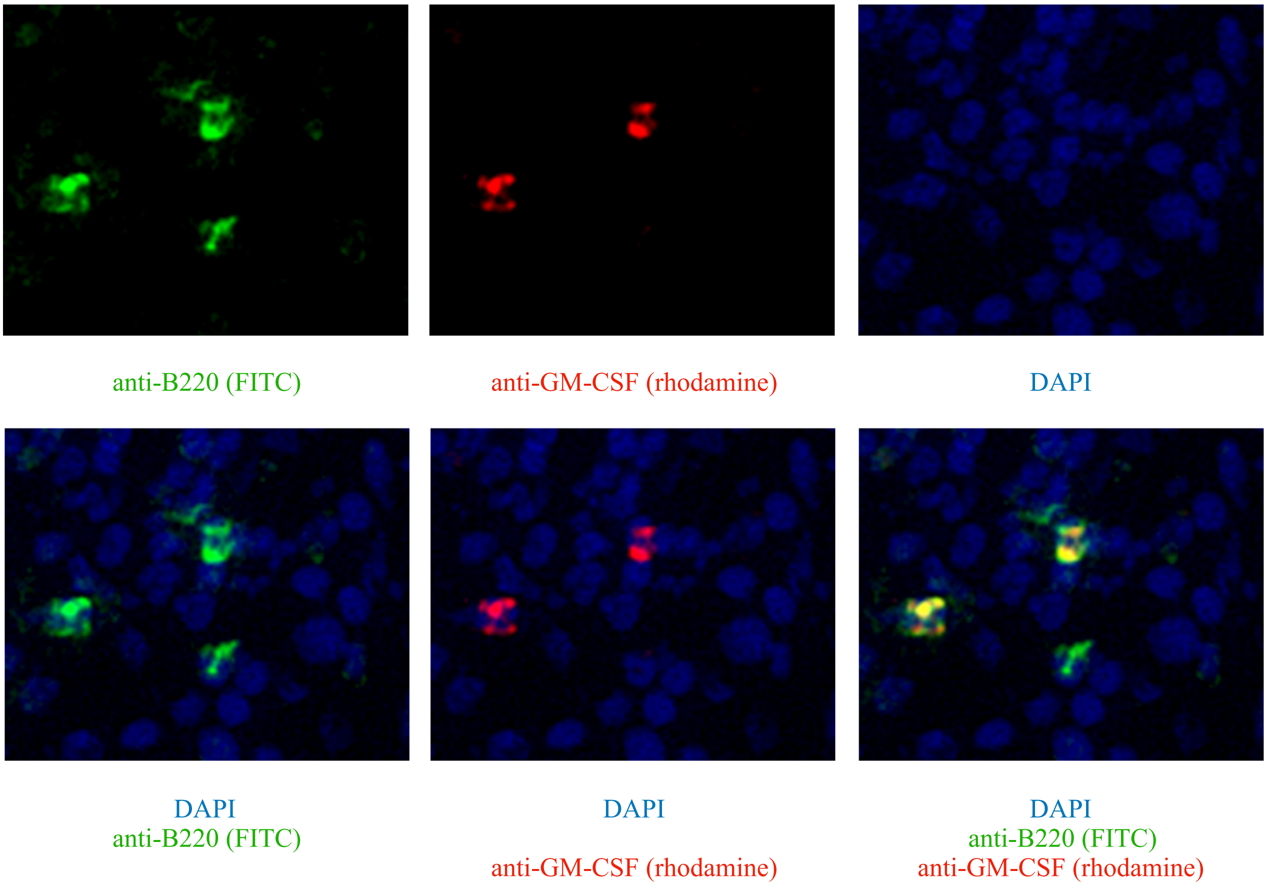

Supplementary Figure 2. Identification of splenic innate response activator (IRA) B cells by immunhistochemistry.

7 days after induction of abdominal sepsis by *colon ascendens stent peritonitis* (CASP), the spleens of the mice were harvested. Sections (3 µm) were stained with anti-B220 (green), anti-GM-CSF (red) and DAPI (blue). IRA B cells were identified as B220^{+} GM-CSF^{+} cells.

Supplementary Table 1: Cytokine quantification in plasma, peritoneal lavage fluid and spleen after CASP induction in wt and $s1pr_4^{-/-}$ mice

Plasma	*wt*		*$s1pr_4^{-/-}$*			
	Median	Range	Median	Range	p-val	
IL-1β	2807	964,1	37,75	749,1	0,0732	ns
IL-2	11,75	2,062	16,13	8,141	0,1714	ns
IL-3	909,8	138	1033	145,5	0,5458	ns
IL-6	85,2	100,5	2182	4149	0,0427	*
IL-10	36,32	59,08	1353	3222	0,0006	***
IL-12p70	n.d.		n.d.			
IL-17A	n.d.		n.d.			
CXCL13	24996	1221	19850	2864	0,2718	ns
GM-CSF	59,61	20,83	61,99	60,8	0,873	ns
IFN-γ	n.d.		n.d.			
MCP-1	43,65	24,03	538,1	2891	0,2817	ns
TGF-β	n.d.		n.d.			
TNF-α	11,14	6,064	35,98	46,82	0,0952	ns

Lavage	*wt*		*$s1p_4^{-/-}$*			
	Median	Range	Median	Range	p-val	
IL-1β	42,03	35,56	44,38	24,99	0,7164	ns
IL-2	20,74	4,578	16,71	7,047	0,832	ns
IL-3	955,5	266,9	211,6	97,59	0,0977	ns
IL-6	1442	1181	3369	2000	0,1884	ns
IL-10	49,37	52,36	1037	277,7	0,0068	**
IL-12p70	8,07	1,025	7,28	2,385	0,5667	ns
IL-17A	5,57	4,043	80,76	58,04	0,0734	ns
CXCL13	3908	1918	1647	1383	0,1725	ns
GM-CSF	94,91	13,94	39,08	23,85	0,2967	ns
IFN-γ	7,19	1,81	9,42	5,212	0,5253	ns
MCP-1	77,71	191,9	311,1	518,6	0,0943	ns
TGF-β	21,04	3,94	22,39	8,847	0,9999	ns
TNF-α	23,57	2,863	30,96	7,228	0,4394	ns

Spleen	*wt*		*$s1p_4^{-/-}$*			
	Median	Range	Median	Range	p-val	
IL-1β	28,54	151	39,07	156,8	0,8507	ns
IL-2	36,3	9,967	36,8	9,677	0,885	ns
IL-3	659,6	63,37	667,7	79,65	0,536	ns
IL-6	14,6	5,221	113,7	395,3	0,1025	ns
IL-10	25,59	25,32	125,7	171	0,1296	ns
IL-12p70	n.d.		n.d.			
IL-17A	n.d.		n.d.			
CXCL13	5068	1358	3435	1462	0,2423	ns
GM-CSF	48,89	35,8	22,49	27,2	0,373	ns
IFN-γ	13,14	3,055	26,28	20,25	0,3517	ns
MCP-1	10,15	4,931	69	46,81	0,1128	ns
TGF-β	374,4	51,93	277,7	85,2	0,8244	ns
TNF-α	9,845	9,723	37,22	9,836	0,2	ns

n.d. = non-detectable

Supplementary Table 2: List of antibodies used.

**Name**	**Isotype**	**Clone**	**Concentration**	**Dilution**	**Manufacturer**
anti-CD11b-APC eFluor 780	Rat IgG2b, κ	M1/70	0,2 mg/ml	1:1000	eBioscience (Affymetrix), Santa Clara, CA, USA
anti-CD19-eFluor 660	Rat IgG2a, λ	93	0,5 mg/ml	1:500	BioLegend, San Diego, CA, USA
anti-CD23-Biotin	Rat IgG2a, κ	B3B4	0,5 mg/ml	1:100	BioLegend, San Diego, CA, USA
anti-CD23-PE	Rat IgG2a, κ	eBio1D3	0,2 mg/ml	1:200	eBioscience, Santa Clara, CA, USA
anti-CD3-FITC	Armenian Hamster IgG	145-2C11	0,5 mg/ml	1:100	eBioscience, Santa Clara, CA, USA
anti-CD3-BV605	Armenian Hamster IgG	145-2C11	0,2 mg/ml	1:100	BioLegend, San Diego, CA, USA
anti-CD4-BV421	Rat IgG2b, κ	GK1.5	25 µg/ml	1:40	BioLegend, San Diego, CA, USA
anti-CD45R (B220)-Alexa647	Rat IgG2a, κ	RA3-6B2	0,5 mg/ml	1:20	Miltenyi Biotec, Bergisch Gladbach, DE
anti-CD45R (B220)-Biotin	Rat IgG2a, κ	RA3-6B2	0,5 mg/ml	1:20	Miltenyi Biotec, Bergisch Gladbach, DE
anti-CD45R (B220)-FITC	Rat IgG2a, κ	RA3-6B2	0,15 mg/ml	1:20	Miltenyi Biotec, Bergisch Gladbach, DE
anti-CD5-PE/Cy7	Rat IgG2a, κ	53-7.3	0,2 mg/ml	1:200	BioLegend, San Diego, CA, USA
anti-FITC-Alexa488	Rabbit IgG Fraction	A11090	1 mg/ml	1:100	life technologies, Carlsbad, CA, USA
anti-GL7-Biotin	Rat IgG2a, κ	GL-7	0,5 mg/ml	1:50	eBioscience, Santa Clara, CA, USA
anti-GM-CSF-Biotin	Rat IgG2a, κ	554407	0,5 mg/ml	1:100	BD Biosciences, San Jose, CA, USA
anti-IgA-BV421	Rat IgG2a, κ	C10-1	0,2 mg/ml	1:100	BD Biosciences, San Jose, CA, USA
anti-IgD-V450	Rat IgG2a, κ	11-26c.2a	0,2 mg/ml	1:100	BD Biosciences, San Jose, CA, USA

Name	Isotype	Clone	Concentration	Dilution	Manufacturer
anti-IgD-Biotin	Rat IgG2a, κ	MD78Z	0,5 mg/ml	1:100	Southern Biotech, Birmingham, AL, USA
anti-IgM-BV650	Rat IgG2a, κ	R6-60.2	0,2 mg/ml	1:100	BD Biosciences, San Jose, CA, USA
anti-IgM-FITC	Rat IgG2a, κ	II/41	0,5 mg/ml	1:100	Invitrogen, Carlsbad, CA, USA
anti-IgM-Biotin	Rat IgG2a, κ	RTK2118	0,5 mg/ml	1:100	BioLegend, San Diego, CA, USA
anti-MAdCAM-1 Alexa488	Rat IgG2a, κ	MECA-367	0,5 mg/ml	1:50	BioLegend, San Diego, CA, USA
anti-Rb-IgG-BV421	Donkey IgG	Poly4064	0,1 mg/ml	1:50	BioLegend, San Diego, CA, USA
anti-Rb-IgG-FITC	Goat IgG	ab6717	2 mg/ml	1:100	Abcam, Cambridge, UK
Fc-Block (TrueStain FcX® CD16/32)	Rat IgG2a, λ	93	0,5 mg/ml	1:100	BioLegend, San Diego, CA, USA
IgG-Alexa488 (Isotype control)	Rat IgG2a, κ	eBR2a	0,5 mg/ml	1:100	eBioscience, Santa Clara, CA, USA
IgG-APC (Isotype control)	Rat IgG2a, κ	eBR2a	0,2 mg/ml	1:250	eBioscience, Santa Clara, CA, USA
IgG-Biotin (Isotype control)	Rat IgG2a, κ	eBR2a	0,5 mg/ml	1:100	eBioscience, Santa Clara, CA, USA
IgG-BV421 (Isotype control)	Rat IgG2a, κ	MOPC-173	0,5 mg/ml	1:100	BioLegend, San Diego, CA, USA
DAPI				1:1000	Molecular Probes, Eugene, OR, USA
Draq5™				1:500	BioLegend, San Diego, CA, USA
Fluorescein-labeled PNA				1:500	Vector Laboratories, Burlingame, CA, USA
Rhodamine (TRITC)-conjugated Streptavidin				1:500	Jackson ImmunoResearch Laboratories Inc., West Grove, PA, USA

Supplementary Table 3: Primer sequences used for quantification of Sphingosine-1-phosphate (S1P) receptor subtypes and β_2-microglobulin.

**Primer**	**Sequence**
$S1P_1$ forward	5′-AAC TTT GCG AGT GAG CTG GT-3′
$S1P_1$ reverse	5′-CTA GAG GGC GAG GTT GAG TG-3′
$S1P_2$ forward	5′-AAC TCC GGG ACA TAG ACC GA-3′
$S1P_2$ reverse	5′-GGC TGA GCA CTG GCT AGG-3′
$S1P_3$ forward	5′-GCC CCT AGA CGG GAG TCT TA-3′
$S1P_3$ reverse	5′-ATA GGC TCT CGT TCT GCA AAG-3′
$S1P_4$ forward	5′-GGA CTT CTC GGT CAC TCA GC-3′
$S1P_4$ reverse	5′-GGC TTG CTG TCA TGT TCT CA-3′
$S1P_5$ forward	5′-GGA GGG ACT CTC CTG GAT TC-3′
$S1P_5$ reverse	5′-TTC CTC TGT AGC CAG CCA CT-3′
β_2-microglobulin forward	5′-ATT CAC CCC CAC TGA GAC TG-3′
β_2-microglobulin reverse	5′-GCT ATT TCT TTC TGC GTG CAT-3′
